# Fluorescence‐ and bioluminescence‐based approaches to study GPCR ligand binding

**DOI:** 10.1111/bph.13316

**Published:** 2015-11-05

**Authors:** Leigh A Stoddart, Carl W White, Kim Nguyen, Stephen J Hill, Kevin D G Pfleger

**Affiliations:** ^1^ Cell Signalling Research Group, School of Life SciencesUniversity of NottinghamNottinghamUK; ^2^ Molecular Endocrinology and PharmacologyHarry Perkins Institute of Medical ResearchNedlandsWAAustralia; ^3^ Centre for Medical ResearchThe University of Western AustraliaCrawleyWAAustralia

## Abstract

Ligand binding is a vital component of any pharmacologist's toolbox and allows the detailed investigation of how a molecule binds to its receptor. These studies enable the experimental determination of binding affinity of labelled and unlabelled compounds through kinetic, saturation (*K_d_*) and competition (*K_i_*) binding assays. Traditionally, these studies have used molecules labelled with radioisotopes; however, more recently, fluorescent ligands have been developed for this purpose. This review will briefly cover receptor ligand binding theory and then discuss the use of fluorescent ligands with some of the different technologies currently employed to examine ligand binding. Fluorescent ligands can be used for direct measurement of receptor‐associated fluorescence using confocal microscopy and flow cytometry as well as in assays such as fluorescence polarization, where ligand binding is monitored by changes in the free rotation when a fluorescent ligand is bound to a receptor. Additionally, fluorescent ligands can act as donors or acceptors for fluorescence resonance energy transfer (FRET) with the development of assays based on FRET and time‐resolved FRET (TR‐FRET). Finally, we have recently developed a novel bioluminescence resonance energy transfer (BRET) ligand binding assay utilizing a small (19 kDa), super‐bright luciferase subunit (NanoLuc) from a deep sea shrimp. In combination with fluorescent ligands, measurement of RET now provides an array of methodologies to study ligand binding. While each method has its own advantages and drawbacks, binding studies using fluorescent ligands are now a viable alternative to the use of radioligands.

**Linked Articles:**

This article is part of a themed section on Molecular Pharmacology of G Protein‐Coupled Receptors. To view the other articles in this section visit http://onlinelibrary.wiley.com/doi/10.1111/bph.v173.20/issuetoc

**Abbreviations**
APEC: 2‐[[2‐ [4‐[2‐(2‐aminoethyl)‐aminocarbonyl]ethyl]phenyl]ethylamino]‐5′‐N‐ethyl carboxamidoadenosine
BODIPY: boron‐dipyrromethene
BRET: bioluminescence resonance energy transfer
FA: fluorescence anisotropy
FFA_1_: free fatty acid receptor 1
FP: fluorescence polarization
TR‐FRET: time‐resolved fluorescence resonance energy transfer

## Tables of Links

**Table:** 

**TARGETS**	
**GPCRs**	
5‐HT2C receptor	FFA1 (free fatty acid) receptor
Adenosine A1 receptors	Formyl peptide receptors
Adenosine A2A receptor	Ghrelin receptors
Adenosine A3 receptors	Melanocortin MC4 receptor
β1‐adrenoceptors	Muscarinic M1 receptors
β2‐adrenoceptors	Neuropeptide Y receptor
Angiotensin AT1 receptors	δ opioid receptors
Cholecystokinin CCK1 receptors	PTH1 receptor
CXCR4	Vasopressin V1A receptors
Dopamine D2 receptors

**Table:** 

**LIGANDS**
AB‐MECA
CGP 12177
ICI 118551
MSH
Propranolol
PTH, parathyroid hormone
SCH442416

These Tables list key protein targets and ligands in this article which are hyperlinked to corresponding entries in http://www.guidetopharmacology.org, the common portal for data from the IUPHAR/BPS Guide to PHARMACOLOGY (Pawson *et al*., 2014) and are permanently archived in the Concise Guide to PHARMACOLOGY 2013/14 (Alexander *et al*., 2013).

## Introduction

**Figure 1:**
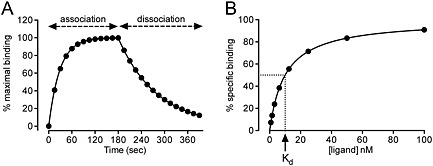
Simulated kinetic and saturation binding curves. Typical kinetic and saturation binding curves were simulated in GraphPad Prism using the association and dissociation kinetics equation (A) or one‐site specific binding saturation equation (B) for a ligand with a 10 nM *K_d_* for a receptor. This demonstrates the difference in the data that can be generated for the same ligand depending on the assay format used. For (A), the concentration of ligand was set to 25 nM, *k*_on_ to 1 x 10^6^ M^−1^·s^−1^ and *k*_off_ to 0.01 M^−1^, which gives a *K_d_* of 10 nM. In (B), the *B*_max_ was set to 100 and *K_d_* to 10 nM and represents total minus non‐specific binding (as determined in the presence of a high concentration of unlabelled ligand). The *K_d_* is equivalent to the concentration of ligand resulting in 50% of specific binding.

**Figure 2:**
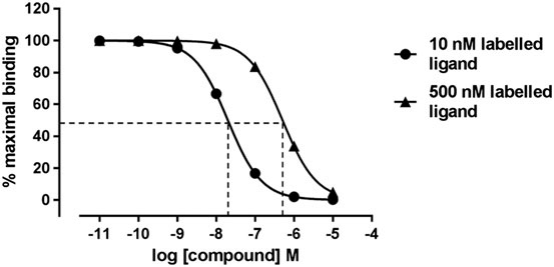
Simulated competition binding curves using two different concentrations of labelled ligand. Typical competition binding curves were generated in GraphPad Prism. The one site competition equation (which incorporates the Cheng–Prusoff correction) was used with 10 nM and 500 nM of the labelled ligand, which had a *K_d_* of 10 nM. The IC_50_ values for these curves were 20 nM and 500 nM, respectively. Using the Cheng–Prusoff equation, this gives a *K_i_* of 10 nM for the unlabelled ligand under both conditions. This demonstrates the differences in the concentration–response curves and IC_50_ values that can be obtained if high concentrations of the labelled ligand are used.

One of the most powerful tools in pharmacology is the ability to measure how well a molecule of interest binds to a protein. This measure of binding, or affinity, can be derived in many different ways depending on the class of protein studied. G protein‐coupled receptors (GPCRs) are the largest family of receptor proteins in the human genome (Hill, 2006). The affinity of molecules for a GPCR can be measured in two main ways, either through detection of a molecule that has been labelled in some way or in a functional assay to quantify the effect of an antagonist, such as Gaddum and Schild analyses (Gaddum, 1957; Schild, 1947). Here, we will focus on the techniques that measure the levels of labelled ligand. This labelling has historically been achieved through the use of radioisotopes utilized in binding assays, of which there are three main types of experimental protocol that allow affinity to be determined: kinetic, saturation and competition (Figures 1 and 2). Due to safety concerns associated with exposure to radioactivity and the costs associated with licencing and disposal, a viable labelling substitute has been sought. For many GPCR researchers, the most promising alternative is through the fluorescent labelling of pharmacologically active molecules (Sridharan *et al*., 2014; Ward and Milligan, 2014). However, it is important to keep in mind that the incorporation of a fluorescent label can markedly change the structure of the molecule and, especially for small molecule ligands (MW of <600), this means that the fluorescent ligand can have different interactions with the receptor and therefore should be considered distinct from the parent compound.

The aim of every binding assay for a GPCR is to quantify the levels of bound ligand. For radioligand binding assays, this is achieved by scintillation counting; however, quantitation of the binding of fluorescent ligands to GPCRs required new developments in plate reader technology and assay formats. This review will first briefly cover the theory behind determining the affinity of compounds experimentally at a GPCR and then discuss the variety of different fluorescence‐based and bioluminescence‐based approaches that have been taken to measure ligand binding.

## Experimental determination of affinity

Determination of the affinity of a labelled or unlabelled compound is based on the fitting of experimental data to a mathematical model. An in‐depth discussion of the mathematical equations used and the theory behind them is beyond the scope of this present review and can be found in pharmacology textbooks such as ‘A Pharmacology Primer’ (Kenakin, 2009) or within other reviews (Hulme and Trevethick, 2010; Motulsky and Neubig, 2010). Nonetheless, some of the basic principles behind each of these assays need to be taken into consideration before discussing how these have been applied using fluorescent ligands. Firstly, the reversible binding of a molecule to a receptor should conform to the law of mass action:

$$L+R\overset{k_{\text{on}}}{\underset{k_{\text{off}}}{⇄}}LR$$

Where *L* is the ligand or molecule, *R* is the receptor, *LR* is the ligand receptor complex, *k*_on_ is the association rate constant and *k*_off_ is the dissociation rate constant. The rate of binding (association) of ligand is given by *k*_on_ × [*L*] × [*R*], whilst the rate of dissociation is given by *k*_off_ × [*LR*], where [*L*], [*R*] and [*LR*] are the free concentrations of *L*, *R* and *LR* at a particular time point. If this reaction is allowed to progress to equilibrium, which is when the rate of dissociation is equal to the rate of association, then under these conditions the equilibrium dissociation constant (*K_d_*) can be determined as follows:

$$K_{d}=\frac{\left[R\right]\times \left[L\right]}{\left[LR\right]}=\frac{k_{\text{off}}}{k_{\text{on}}}$$

The *K_d_* is a measure of the affinity of a ligand for a receptor and is the concentration of ligand required to bind 50% of the total receptor binding sites at equilibrium (i.e. when [*LR*] equals [*R*]). With kinetic binding experiments, experimental measurement of the association and dissociation rate of a fixed concentration of labelled ligand allows the *K_d_* to be calculated (Figure 1A). Similarly, the *K_d_* can be calculated from the observed association kinetic data as follows:

$$k_{obs}=K_{\text{on}}\times \left[L\right]+K_{\text{off}}$$

Where *k*_obs_ is calculated from global fitting of data to the following monoexponential association function using data generated with multiple known values of [*L*]:

$$Y=Y_{\text{max}}.\left(1-e^{-k_{obs}.\;t}\right)$$

*Y*_max_ is the binding at infinite time (*t*), and *k*_obs_ is the rate constant for the observed rate of association. This enables both *k*_on_ and *k*_off_ to be shared between the curves generated for different values of [*L*] and yield a *K_d_* from their ratio.

To overcome the difficulties associated with kinetic experiments using radioligands, and the requirement to separate bound ligand from free ligand at every time point, saturation binding experiments can also be used to determine *K_d_*. A saturation binding assay uses increasing concentrations of labelled ligand incubated with the receptor population of interest until equilibrium is reached and then measures the levels of bound labelled ligand (Figure 1B). In this situation, the level of specifically bound ligand is defined by the following:

$$\text{Specific Binding}\;=\;\frac{B_{\text{max}}\;\times \;\left[L\right]}{\left[L\right]+K_{d}}$$

Where *B*_max_ is the maximal specific binding, and the other parameters are as described previously. In this experimental set‐up, the free labelled‐ligand concentration is assumed to be identical to that originally added. That is, the receptor concentration is too low to change the free labelled‐ligand concentration. In addition, a parallel set of conditions is normally included in the presence of a fixed high concentration of unlabelled ligand in order to determine the binding of the labelled ligand to non‐specific sites. As the name suggests, saturation of the specific binding sites should be reached where no additional binding is seen with increasing concentration of labelled ligand. If saturation is reached and the concentration of the labelled ligand can be accurately determined, an estimation of the available binding sites can also be calculated (*B*_max_). Due to the limitations of using the large amount of radioactivity required to reach saturation, these experiments are normally carried out using concentrations of radioligand spanning no more than about two orders of magnitude and are plotted on a linear scale.

It is not viable to label every molecule for which the affinity is to be determined. For compounds labelled with tritium (^3^H) this is theoretically possible as the structure of the molecule is unchanged, but this would require large amounts of radioactivity. To measure the affinity of unlabelled ligands, a third type of binding experiment can be performed termed a competition binding assay. The principle behind this experimental approach is that increasing concentrations of an unlabelled ligand will compete with a fixed concentration of a labelled ligand for the same receptor binding sites. In the basic form, comparison of the concentration of unlabelled compounds required to inhibit by 50% the specific binding of the labelled ligand (IC_50_) can be used to generate a rank order of potencies. However, it is important to note that the exact IC_50_ obtained will depend on the concentration of the labelled ligand used. Higher concentrations of competing ligand will be required to inhibit the binding of a higher concentration of labelled ligand (Figure 2). If the affinity of the labelled ligand is known, then the Cheng–Prusoff equation (Cheng and Prusoff, 1973) can be applied to address this:

$$K_{i}=\frac{IC_{50}}{1+\frac{\left[L\right]}{K_{d}}}$$

This equation takes into account the concentration ([*L*]) and *K_d_* of the labelled ligand.

These equations for calculating the affinity of labelled and unlabelled compounds were derived from experimental data performed with radiolabelled ligands, but if the theory and assumptions behind each of the equations are taken into consideration, then they can be used with fluorescently labelled ligands. The main assumptions are that the binding of both the labelled and unlabelled ligand is reversible and that they compete for a similar binding site in a receptor, whether it is the orthosteric or an allosteric site. These assumptions are rarely achieved as both membrane and whole cell binding assays present different challenges in meeting these conditions. With the use of membranes, both the extracellular and intracellular faces of the receptor are exposed to the same concentration of ligands, and some ligands may preferentially bind to intracellular binding sites that may not be available in a whole cell system. On the other hand, in a whole cell system, both fluorescent and radioligands can be taken up into the cell, especially if they are lipophilic, which can lead to dificulties in demonstrating that the binding is reversible and also lead to high levels of non‐specific binding. In addition, the equations for saturation and competition binding assays are based on the assumption that the assays are performed at equilibrium. One way to confirm that the experiment is at equilibrium is to perform the assays at different time points, and if there is a change in the *K_d_* or *K_i_* values with increasing incubation times, then the assay is not at equilibrium. Another assumption is that a small fraction of the added ligand (less than 10%) is bound to receptor binding sites so that the free concentration in a solution approximates to that added. Otherwise, the situation known as ligand depletion becomes appreciable and can have a significant effect on calculated affinity values in both saturation and competition assays. A major consideration in developing a robust binding assay is the efficacy of the labelled compound. In general, antagonist molecules are preferred as they label a single population of receptors (i.e. do not distinguish between receptor conformations R and R^*^), whereas an agonist will bind to both R and R^*^ conformations of the receptor with differing affinities, and this often leads to biphasic curves. The use of a labelled agonist in a whole cell competition binding system will activate the receptor and lead to receptor internalization. This will remove a portion of the receptors from the cell surface and prevent the competing ligand accessing the whole population of receptors, which may result in variations in affinity values when compared with binding with a labelled antagonist.

## Direct measurement of fluorescence

### Fluorescence polarization/anisotropy

Some of the earliest examples of using fluorescent ligands to monitor binding to GPCRs used fluorescence anisotropy (FA) (Tota *et al*., 1994; Tota *et al*., 1995; Turcatti *et al*., 1995). FA, which can also be referred to as fluorescence polarization (FP) depending on the equation used, is based on measuring the ability of a fluorescent molecule to maintain the polarization of light. If not bound to a receptor and free in solution, the free rotation of the fluorescent ligand means that the direction of the emitted light will not be at the same angle as the polarized light used to excite the fluorophore. Whereas, if the ligand is bound to a receptor and is therefore in a fixed position, the light will be emitted in the same plane as the original excitation and maintain the polarization. This difference in polarization can be used to differentiate bound from free ligand. The early examples used a cuvette‐based spectrofluorometer and were therefore very low throughput (Tota *et al*., 1994; Tota *et al*., 1995; Turcatti *et al*., 1995), but advances in plate reader technology has led to FA and FP experiments being performed in 96‐ (Cornelius *et al*., 2009; Kecskes *et al*., 2010; Veiksina *et al*., 2010; Harikumar *et al*., 2011), 384‐ (Allen *et al*., 2000) and even 1536‐ (Harris *et al*., 2003) well plate formats. A range of different GPCRs, both peptide and non‐peptide, has been successfully studied using FP experiments. The GPCRs with low MW endogenous ligands include the 5‐HT_2C_ receptor (Cornelius *et al*., 2009), adenosine A_2A_ receptor (Kecskes *et al*., 2010) and muscarinic M_1_ receptor (Harris *et al*., 2003), and the peptide receptors include the cholecystokinin CCK_1_ receptor (Harikumar *et al*., 2011), vasopressin V_1A_ and δ opioid receptors (Allen *et al*., 2000). As FA and FP can distinguish between bound and unbound ligand, there is no requirement for a wash or filtration step. It is therefore a homogenous assay and is normally performed on cell membranes. Due to its homogenous nature, experiments can theoretically be undertaken using FA or FP to measure the kinetic parameters of ligand binding. However, it should be pointed out that a high degree of ligand depletion is normally a consequence of the high receptor concentrations and low ligand concentrations required to obtain a significant change in FA/FP on ligand binding. As a consequence, there is the risk of significant changes in the free concentrations of both labelled and unlabelled ligands during kinetic and competition binding experiments with this technique. Nevertheless, kinetic assays have been successfully undertaken for the adenosine A_2A_ receptor using a fluorescent antagonist, MRS5346, which comprised the A_2A_ selective antagonist SCH442416 linked to the Alexa Fluor‐488 fluorophore. The *K_d_* determined kinetically for the fluorescent ligand differed from that calculated from a competition radioligand binding assay using an agonist radioligand (16.5 ± 4.7 nM vs. 111 ± 16 nM) (Kecskes *et al*., 2010), supporting the fact that agonists and antagonist can label different conformations of the receptor leading to differences in the derived affinity values as discussed under ‘Experimental determination of affinity’. Kinetic studies have also been performed using the melanocortin MC_4_ receptor and a fluorescent derivative of the agonist MSH, which allowed estimation of equilibrium times to be determined, and in this case, equilibrium was not reached until 3 h (Veiksina *et al*., 2010). FA and FP studies have also been used to estimate the IC_50_ of unlabelled compounds. Furthermore, where the levels of fluorescent ligand used have been taken into account and the Cheng–Prusoff equation applied, *K_i_* values have been calculated. Where *K_i_* values have been determined, these appear to correlate well with those obtained by radioligand binding (Cornelius *et al*., 2009; Kecskes *et al*., 2010). As mentioned earlier, a potential major disadvantage of using FA and FP assays to study ligand binding at GPCRs is the possibility of ligand depletion in competition binding assays. The signal‐to‐noise ratio for FP and FA assays is relatively small and decreases with higher concentrations of ligand; therefore, many assays are performed at low ligand concentrations with high proportion of the ligand bound to the receptor, with up to 20% of the ligand bound in some cases (Allen *et al*., 2000). This may have a significant effect on the measured affinity of both labelled and unlabelled compounds. Ligand depletion may be a particular issue for kinetic studies as the saturation of signal could be prematurely reached and therefore give an artificially fast association rate.

### Confocal microscopy‐based ligand binding

Although it has been suggested that direct measurement of fluorescent ligand binding to a receptor is not possible due to the cellular auto‐fluorescence and sensitivity issues (Cottet *et al*., 2011), the direct imaging and quantification of levels of bound fluorescent ligand have been successfully applied in both competition (Stoddart *et al*., 2012; Vernall *et al*., 2013; Gherbi *et al*., 2014) and kinetic binding assays (May *et al*., 2010; May *et al*., 2011; Gherbi *et al*., 2015). The direct imaging of a fluorescent ligand is usually achieved through the use of confocal microscopy. With fluorophores that emit in the red or far‐red range of the spectrum, difficulties arising from auto‐fluorescence are reduced. One of the first examples of the use of confocal microscopy to quantify the binding of a fluorescent ligand to a receptor of interest was performed on live cells expressing the β_2_‐adrenoceptor and a fluorescent derivative of the partial agonist CGP 12177 [boron‐dipyrromethene (BODIPY)‐TMR‐CGP] (Baker *et al*., 2003). Individual confocal images were obtained manually with increasing concentrations of the fluorescent ligand. Clear membrane binding of the fluorescent ligand was observed, and a saturation binding curve was generated using average pixel intensity from the images. This yielded a *K_d_* value similar to that obtained in radioligand binding studies (Baker *et al*., 2003). However, it is worth mentioning here that a large concentration range of fluorescent ligand cannot be used in these experiments, as the dynamic range of a confocal microscope is not as large as that of a scintillation counter. In addition, the reversibility of the binding was shown with increasing concentrations of unlabelled ligands, which enabled the generation of competition curves and *K_i_* values similar to those previously observed (Baker *et al*., 2003). This study demonstrated the potential of direct monitoring of fluorescent ligand binding, but the manual nature of obtaining the images makes it time‐consuming to generate even a simple concentration–response curve. To overcome the low‐throughput aspect of this method, a high content screening confocal microscopy‐based system has been used in place of a standard confocal microscope. This allowed the capture and processing of hundreds of images from a multiwell plate automatically. It was first applied to adenosine A_1_ and A_3_ receptors using a xanthine amine congener (XAC)‐based fluorescent ligand (CA200645), which does not discriminate between the two receptor subtypes. Again, these experiments were performed in live cells and at 37°C. Clear competition of the fluorescent ligand with unlabelled ligands was observed in the images, and the calculated *K_i_* values were in close agreement with those obtained in different assays. In addition, using receptor selective compounds, the expected pharmacology was observed, confirming that the loss in signal with competing compounds was specific and not an artefact of the experimental approach (Stoddart *et al*., 2012).

This system has also recently been applied to the β_1_‐adrenoceptor, again using BODIPY‐TMR‐CGP as the fluorescent ligand. Through the use of labelled CGP 12177, the ability of the ligand to bind to both the classical catecholamine binding site of the β_1_‐adrenoceptor and to a secondary low‐affinity receptor conformation was confirmed (Gherbi *et al*., 2014). Importantly, the use of fluorescently labelled CGP 12177 allowed high concentrations of the ligand to be tested that, although not impossible, would be more challenging to use with a radioligand due to cost and safety issues.

In addition to saturation and competition‐type experiments, the direct visualization of fluorescent ligand binding to GPCRs has been used to study the kinetics of ligand binding in whole, live cells at physiological temperature. The use of a confocal microscope coupled to a closed perfusion system that ensured constant flow of buffer over the cells enabled the controlled addition and removal of fluorescent ligands and unlabelled drugs. This allowed measurement of the kinetic parameters of a fluorescent agonist (ABEA‐X‐BY630) at the adenosine A_1_ and A_3_ receptors at the single cell level. The advantage of using BODIPY fluorophore‐linked fluorescent ligands is that they are heavily quenched in aqueous solution and are brighter in a non‐aqueous environment, that is, when receptor bound (Baker *et al*., 2010). This means that separation of bound from free ligand is not necessary, and association rates can be measured in real time. In keeping with receptor theory, the observed *k*_obs_ increased with increasing concentrations of fluorescent ligand, and there was no change in *k*_off_. The calculated *K_d_* was likely to represent binding of the ligand to the high affinity active conformation of the receptor (R*) because low concentrations of fluorescent agonist were used in these experiments. In addition to the direct measurement of the kinetics of the labelled ligand, the effect of allosteric modulators on the dissociation of the fluorescent agonist can be studied in the absence of additional unlabelled ligand as the constant buffer flow removes the unbound or dissociated ligand (May *et al*., 2010). This removal of unbound ligand is referred to as infinite dilution, which is also possible through the addition of a large volume (100x assay volume) of buffer (Christopoulos *et al*., 1997). An equivalent experiment to study dissociation rates is more often used with radioligands, as the large dilution factor required for infinite dilution can be experimentally challenging. It relies on the addition of a high concentration of unlabelled orthosteric ligand to prevent rebinding of the labelled ligand, based on the assumption that the orthosteric ligand has no influence on the kinetics of the labelled ligand. For the adenosine A_3_ receptor, further studies using ABEA‐X‐BY630 indicated that orthosteric ligands had a profound effect on the dissociation rate of the labelled ligand as a consequence of negative cooperativity across a homodimer interface (May *et al*., 2011), indicating that the assumption that the unlabelled ligand has no effect is not always true. Overall, the direct measurement of fluorescent ligands by confocal microscopy can be a powerful tool to perform competition and kinetic binding assays.

### Flow cytometry

Flow cytometry measures the mean fluorescence intensity of each cell that passes through the path of a flow cytometer. When fluorescent ligands are available, this technique can be used for ligand binding studies of single live cells. A relatively high signal to background ratio can be obtained due to the relatively small fluid volume excited per single cell by the cytometer laser and if the fluorescent ligand has a significantly lower quantum yield in free solution compared with ligand bound to receptor (in a lipid environment). Under these circumstances, ligand binding data can be collected in real‐time on a microsecond timescale without the need to remove unbound ligands from the media. With the current array of fluorescent ligands, flow cytometry has been used for ligand binding experiments for a number of different GPCRs including adenosine A_3_ receptor (Kozma *et al*., 2012; Corriden *et al*., 2013; Kozma *et al*., 2013), chemokine receptor CXCR4 (Hatse *et al*., 2004), neuropeptide Y receptor (Schneider *et al*., 2007), free fatty acid FFA_1_ receptor (Hara *et al*., 2009) and formyl peptide receptors (Sklar and Finney, 1982; Sklar *et al*., 1984; Sklar *et al*., 1985), as well as the yeast α‐factor receptor (Bajaj *et al*., 2004).

Some of the earliest studies using flow cytometry investigated the kinetic association and dissociation as well as internalization of fluorescein‐labelled formyl peptide to chemotactic receptors on human neutrophils. Additionally, by calibrating the fluorescent signal, these researchers were able to determine the number of binding sites and were able to confirm previous kinetic binding studies using radioligands, as well as provide direct comparison between *K_d_* values calculated by kinetic and saturation binding assays (Sklar *et al*., 1984; Sklar *et al*., 1985). In more recent kinetic binding studies at the adenosine A_3_ receptors, using a modified triazolo‐quinazoline click‐conjugated to Alexa Fluor‐488, the flow cytometry method resulted in a *K_d_* value of 6.65 ± 0.55 nM that was consistent with the equilibrium binding constant of 5.15 ± 1.11 nM calculated by saturation binding experiments in the same study, as well as the *K_i_* of 6.4 ± 2.5 nM calculated by [^125^I]I‐AB‐MECA radioligand competition binding assays (Kozma *et al*., 2012). Although *K_i_* values of adenosine antagonists in competition binding assays with the fluorescent ligand (an antagonist) were consistent with radioligand binding in membranes, *K_i_* values obtained for agonist binding were up to 20‐fold higher than with the radioligand [^125^I]I‐AB‐MECA. Interestingly, in a study by the same group where an adenosine agonist was tethered to Alexa Fluor‐488, the converse was true, that is, *K_i_* values obtained from antagonists were inconsistent with radioligand binding studies (Kozma *et al*., 2013). Competition binding at the FFA_1_ receptor is a unique example of the utility of the flow cytometry technique, as radioligand binding was not possible due to high non‐specific binding of the available radioligands. Purified N‐terminal FLAG‐tagged FFA_1_ receptors were immobilized on G magnetic beads via a FLAG antibody, and BODIPY‐fused free fatty acid analogues were used to determine affinity (*K_d_*) using saturation binding experiments, as well as to perform competition binding in cytometric assays.

One potential advantage of using flow cytometry to study ligand binding at GPCRs is its adaptability to use very low numbers of cells and therefore receptor numbers, helping overcome the possibility of ligand depletion in competition binding assays. However, the use of low cell numbers needs to be balanced with adequate receptor expression and ligand binding. High concentrations of labelled ligand are required to study weak binding, resulting in a high concentration of unbound ligand in the small liquid fraction activated during cytometry. Therefore, the amount of bound labelled ligand needs to be capable of producing fluorescence intensity that is greater than background to result in a detectable signal.

## Resonance energy transfer (RET) methods

### Fluorescence resonance energy transfer (FRET)

FRET utilizes non‐radiative energy transfer from a donor fluorophore to an acceptor fluorophore resulting in fluorescence emission from the acceptor. FRET can occur where there is sufficient overlap of the donor emission spectrum with the acceptor excitation spectrum, close proximity (<100 Å) and correct donor–acceptor orientation of dipole moments. These parameters are readily adaptable to ligand binding assays, where binding of a fluorescent ligand with suitable spectral properties to a fluorophore‐tagged GPCR provides the close proximity required for FRET. Advantageously, in ligand binding assays, non‐specific binding of the ligand to the plasma membrane fails to generate a FRET signal due to the lack of a donor. Practically this means, if designed carefully, that assays can be homogeneous and therefore suitable for kinetic binding assays. In contrast, as the signal observed in these experiments is the result of the efficiency of FRET as well as the amount of ligand bound to tagged receptor, calculation of receptor density is not possible.

FRET assays require the GPCR to be tagged with a donor or acceptor fluorophore, this can be achieved by fusing the N‐terminus of a GPCR with a fluorescent protein such as eGFP or enhanced yellow fluorescent protein. FRET competition binding assays using a BODIPY‐pirenzepine probe and an eGFP‐tagged muscarinic ACh M_1_ receptor have been used to determine the binding affinities of unlabelled muscarinic receptor antagonists (Ilien *et al*., 2003). A constant flow perfusion model, and therefore homogenous conditions, has also been used for FRET experiments. These enabled assessment of ligand binding kinetics of an adenosine receptor agonist, APEC conjugated to Alexa Fluor‐532, at a cyan fluorescent protein‐tagged adenosine A_2A_ receptor (Fernandez‐Duenas *et al*., 2012). Interestingly, the association rate could be allosterically modulated by activation of the dopamine D_2_ receptor oligomerized with the adenosine A_2A_ receptor.

Additionally, non‐covalent labelling of the receptor with a fluorescent antibody against an epitope tag or native sequence can also be used (Albizu *et al*., 2007; Hu *et al*., 2008). However, these strategies are not without their drawbacks: Fluorescent proteins can have low signal‐to‐noise ratios due to wide excitation and emission ranges; auto‐fluorescence of the sample preparation can occur at the acceptor emission wavelength; and there is potential for direct excitation of the acceptor by the light source. Moreover, antibody binding is non‐covalent and has its own binding equilibrium; therefore, kinetic and competition binding experiments depend on both ligand and antibody equilibrium.

To overcome many of these problems, time‐resolved FRET (TR‐FRET) assays have been developed that use lanthanides, commonly in complexes with cryptates, as the donor species. Typically europium or terbium cryptates are used, which have a high signal‐to‐noise ratio due to a long‐lasting emission time that allows for an extended period of observation. The time gating of this measurement effectively eliminates autofluorescence from interfering with the experiment. Additionally, lanthanides have pseudo‐Stokes shifts with several emission wavelengths. This leaves good separation of donor and acceptor spectra, thus further enhancing the signal‐to‐noise ratio, as can be seen in saturation binding experiments at the ghrelin receptor where low non‐specific binding is observed and the signal‐to‐noise ratio is over three times larger than that observed using a radioligand (Leyris *et al*., 2011). Lanthanides can be conjugated to antibodies for labelling of GPCRs, although face the same problems (described above) as fluorescently tagged antibodies. Covalent‐tagging technologies such as SNAP‐tag, CLIP‐tag and Halo‐tag can also be utilized to achieve conjugation. SNAP‐tag technology (Kolberg *et al*., 2013) can be used to fuse the N‐terminus of a target GPCR to the relatively small SNAP‐tag moiety. An addition of a lanthanide donor derivatised to the SNAP‐tag substrate results in a covalently bound donor attached to the GPCR. Conveniently, the use of cell‐impermeable substrates ensures only cell surface receptors are labelled. Importantly, the use of the SNAP‐tag strategy with TR‐FRET experiments allows for kinetic, saturation and competition binding assays to be performed. A study of the parathyroid hormone PTH_1_ receptor (Emami‐Nemini *et al*., 2013) provides a detailed protocol for the use of both SNAP‐tagged receptors as well as fluorescent epitope antibodies in ligand binding studies. Using lanthanide‐derivatised PTH analogues, on‐rates could be determined for both SNAP‐tagged and antibody‐tagged PTH receptors. *K_d_* values from saturation experiments were similar between techniques (35.4 ± 4.7 nM vs. 37.1 ± 5.9 nM, respectively, for [C^35^‐Tb^3+^]PTH(1–34)), indicating that provided equilibrium of both antibody and ligand can be reached, *K_d_* values can be reliably calculated with antibody labelling strategies. Additionally, competition binding experiments produced similar *K_i_* values for four unlabelled PTH analogues despite a large difference in signal‐to‐background (S/B) ratios between techniques (S/B of 30 vs. 9 for SNAP‐tagged vs. antibody‐tagged, respectively). An extensive number of receptors and ligands have been tested using SNAP‐lanthanide‐labelled GPCRs. The affinities (*K_d_*) of red fluorophore‐tagged ligands were determined by saturation binding studies for 18 members of the adrenoceptor, angiotensin, chemokine, cholecystokinin, dopamine, tachykinin and opioid receptor families (Zwier *et al*., 2010). Additionally, the affinities (*K_i_*) of an array of unlabelled ligands were calculated with competitive binding assays, which were reported to be consistent with standard radioligand binding assays.

### Bioluminescence resonance energy transfer (BRET)

The same non‐radiative RET as observed with FRET can occur when a luciferase enzyme oxidizing its substrate acts as the donor instead of a fluorophore (Pfleger and Eidne, 2006). This is known as BRET and is a naturally occurring phenomenon observed in some marine organisms. As with FRET, the efficiency of energy transfer and consequent light emission from the acceptor is dependent upon the following: (1) spectral compatibility between the donor and acceptor (i.e. sufficient overlap of donor emission spectrum with acceptor excitation spectrum); (2) the donor and acceptor being orientated in a way that favours energy transfer; and (3) the donor and acceptor being within 10 nm of each other (Dacres *et al*., 2012). By fusing/conjugating the donor and acceptor to molecules of interest, BRET detection enables real‐time monitoring of molecular proximity in live cells, indicative of interactions between the molecules of interest directly or as complexes (Pfleger *et al*., 2006).

*Renilla* luciferase (Rluc), a 36 kDa luciferase isolated from the sea pansy *Renilla reniformis*, and its corresponding substrate coelenterazine, have been used extensively for studying GPCR protein–protein interactions, most notably with point mutations identified by the Gambhir laboratory that improve performance, resulting in ‘Rluc2’ and ‘Rluc8’ (Loening *et al*., 2006; De *et al*., 2007; Kocan *et al*., 2008). Recently, NanoLuc, a small 19 kDa luciferase subunit isolated from the deep sea shrimp *Oplophorus gracilirostris*, has attracted considerable interest due to its intense luminescence when oxidizing its substrate furimazine. It is approximately 150‐fold more luminescent than the aforementioned Rluc on a per mole basis, exhibits high physical stability, and has an emission maximum of 460 nm (Hall *et al*., 2012).

The extensive development of fluorescent ligands over the last decade (Vernall *et al*., 2014) has provided the opportunity to develop a BRET ligand binding assay where they could be used as energy acceptors. However, there are two major considerations: firstly, for detectable RET to occur, the BRET luciferase/substrate combination needs to emit with sufficient intensity within the excitation wavelength range of the chosen fluorophore. As red fluorophores can be distinguished most readily from cellular autofluorescence and are therefore generally considered most desirable for binding assays, this was potentially a problem as luciferases emit largely at blue wavelengths that are sub‐optimal for exciting red fluorophores (Pfleger and Eidne, 2006). Secondly, a BRET ligand binding assay requires extracellular fusion of the BRET donor luciferase to the receptor of interest. Unfortunately, as receptor proteins are synthesized inside the cell and need to be trafficked and inserted into the plasma membrane, appropriate expression of such a fusion protein is not trivial. Both of these issues have been addressed by utilizing NanoLuc. As a consequence of its very high luminescence intensity, NanoLuc, although having an emission peak at 460 nm, has sufficient emission at longer wavelengths to enable transfer of resonance energy to red fluorophores to be readily detected (Stoddart *et al*., 2015). Furthermore, and more importantly, N‐terminally NanoLuc‐tagged GPCRs have been found to express well on the plasma membrane of cells, in stark contrast to N‐terminally Rluc8‐tagged GPCRs (Stoddart *et al*., 2015). The native luciferase from which NanoLuc is derived is secreted by the shrimp in bright luminescence bursts as a defence against predators (Hall *et al*., 2012). Having evolved to be secreted, and therefore pass across plasma membranes, this may have contributed to the difference compared with Rluc8, in addition to its smaller size (Stoddart *et al*., 2015).

**Figure 3:**
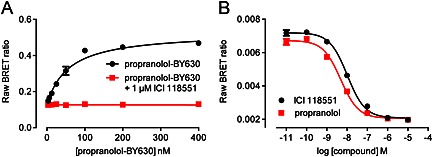
Illustrative NanoBRET saturation and competition binding curves. NanoBRET ligand binding assays were carried out using HEK293 cells stably expressing the human β_2_‐adrenoceptor, as detailed in Stoddart *et al*. (2015). (A) Cells were treated with increasing concentrations of propranolol‐BY630 (compound 18a in Baker *et al*. (2011)) in the presence or absence of 1 μM ICI 118551, resulting in a calculated *K_d_* of 57 nM. (B) Cells were treated with increasing concentrations of ICI 118551 or propranolol and 50 nM propranolol‐BY630, resulting in p*K_i_* values of 8.26 and 8.59, respectively. In both cases, cells were incubated for 1 h at 37°C before the addition of 10 μM furimazine. BY630 fluorescence (>610 nm) and Nanoluc luminescence (420–500 nm) were immediately measured and the fluorescence : luminescence ratio (raw BRET ratio) was calculated. Data shown are representative of three independent experiments and are means ± SEM of triplicate values.

Live cell BRET ligand binding has now been demonstrated using β_2_‐adrenoceptors, adenosine A_1_ and A_3_ receptors, and angiotensin AT_1_ receptors using a range of different fluorescent ligands in saturation, competition and kinetic binding assays (Stoddart *et al*., 2015). Figure 3 shows examples of the saturation and competition binding data that can be generated with the NanoBRET technology at the β_2_‐adrenoceptor. The saturation assays are notable for the multiple orders of magnitude over which labelled ligand concentration can be varied, and BRET lends itself extremely well to real‐time kinetic analysis of ligand‐receptor interactions (Stoddart *et al*., 2015), as would be expected considering the success of previous real‐time analysis of protein–protein interactions using BRET (see Dalrymple *et al*., 2011; Jaeger *et al*., 2014). *K_d_* values generated by saturation and kinetic assays correlated with each other and with values published in the literature using radioligand binding. Furthermore, *K_i_* values calculated for a range of unlabelled ligands competing for these fluorescent ligands were also in good agreement with the literature using radioligands. The BRET ligand binding assay is characterized by a truly homogenous protocol: no conjugation of the receptor to a fluorophore is required as a genetically encoded luciferase‐receptor fusion protein is utilized, and no wash step or lysis is needed. This is because the very high distance dependence of BRET means that separation of free and bound fluorescent ligand is unnecessary.

With the addition of BRET ligand binding to the portfolio of fluorescence‐based ligand binding assays, there are now several alternatives to the use of radioligands for assessing the intricacies of ligand–receptor interactions in live cells. As the range of fluorescent ligands increases, so does the potential of these approaches to play a valuable role in drug discovery, development and profiling in the future, particularly at GPCRs.

## Conflict of interest

Promega Corporation has proprietary rights over the NanoBRET assay and provides funding to K. D. G. P.'s laboratory as a partner organization of ARC Linkage Grant LP130100037.
